# Global analysis of threonine metabolism genes unravel key players in rice to improve the abiotic stress tolerance

**DOI:** 10.1038/s41598-018-27703-8

**Published:** 2018-06-18

**Authors:** Pandiyan Muthuramalingam, Subramanian Radhesh Krishnan, Subramani Pandian, Narayanan Mareeswaran, Wilson Aruni, Shunmugiah Karutha Pandian, Manikandan Ramesh

**Affiliations:** 1 Department of Biotechnology, Science Campus, Alagappa University, Karaikudi, Tamil Nadu 630 003 India; 2 T. Stanes & Company Ltd, Phytopharma Testing Laboratory, Herbal Division, 1128, Coimbatore, 641 018 India; 3 Division of Microbiology, School of Medicine, Loma Linda University, Loma Linda, CA 92350 USA

## Abstract

The diversity in plant metabolites with improved phytonutrients is essential to achieve global food security and sustainable crop yield. Our study using computational metabolomics genome wide association study (cmGWAS) reports on a comprehensive profiling of threonine (Thr) metabolite in rice. Sixteen abiotic stress responsive (AbSR) – Thr metabolite producing genes (ThrMPG), modulate metabolite levels and play a significant role determining both physiological and nutritional importance of rice. These AbSR-ThrMPG were computationally analysed for their protein properties using OryzaCyc through plant metabolic network analyser. A total of 1373 and 1028 SNPs were involved in complex traits and genomic variations. Comparative mapping of AbSR-ThrMPG revealed the chromosomal colinearity with C4 grass species. Further, computational expression pattern of these genes predicted a differential expression profiling in diverse developmental tissues. Protein interaction of protein coding gene sequences revealed that the abiotic stresses (AbS) are multigenic in nature. *In silico* expression of AbSR-ThrMPG determined the putative involvement in response to individual AbS. This is the first comprehensive genome wide study reporting on AbSR –ThrMPG analysis in rice. The results of this study provide a pivotal resource for further functional investigation of these key genes in the vital areas of manipulating AbS signaling in rice improvement.

## Introduction

Plants produce a wide array of biological and chemically altered compounds. Metabolites thus generated play an essential role in their growth, development, and modulate environmental interactions^1,2^. The highest qualitative and quantitative differences in metabolites have made plants as an ultimate model to understand the biosynthetic pathways and functional regulation of metabolites^3,4^. The genetic diversity of plant metabolites and their possible complex regulatory mechanism underlines the importance of investigating the biochemical nature and their fundamentals^5^. These plant metabolites form the principle biochemical base responsible for the crop’s quality and yield, in addition, they are also energy sources and valuable nutrition for human beings and other live stocks^2,5–7^. Metabolites are divided into two major classes, primary and secondary. The primary metabolites are pivotal in the development and growth of a plant; however, secondary metabolites are essential for plants to survive under diverse stress conditions by means of retaining a delicate steadiness with the physiological environment. Furthermore, primary metabolites and their structures are highly conserved and abundant whereas secondary metabolites are extensively diverse down the plant kingdom^8^. Their function in cellular defence against oxidative stress in plants has already been demonstrated^9^. Among the plant metabolites, Threonine (Thr) metabolites have a significant role in chemical defences against abiotic stresses (AbS) such as salt, cold and drought^10–14^. In addition, Thr metabolites are involved in plant growth and development, and cell division, and regulate the phytohormones^13–16^.

Rice (*Oryza sativa* L.) is one of the most significant food crops being consumed by almost half of the world’s population. Understanding the molecular and genetic aspects of its metabolome with their natural variation is vital for the quality, reliability, and sustainability of the world’s food supply. Landraces of *O. sativa* have much evolved from their wild progenitor and show significant genetic diversity^17,18^. Understanding the biochemical basis and genetic features of metabolomics among these different varieties will provide the essential acumen required for developing elite breeds with an improved resistance to destructive stresses and also as a probiotic for humans. To date, evaluating the genetic architecture of rice metabolite traits relied on quantitative trait locus (QTL) with linkage mapping using bi-parental populations^19^. Although this technique provides a better perception^20^, it is unscalable to explore the huge variation in diverse germplasm resources clearly. Importantly, delineating the biosynthetic pathways is traditionally a slow and tedious process^7^. Hence, understanding the genetic and molecular basis of such deviation at the metabolic level is crucial^3,21^.

Genome wide association study combined with metabolomics analysis (or mGWAS) is used to screen a large number of accessions concurrently to delineate genetic contributions to metabolite diversity and their significance to molecular complex traits^22,23^. mGWAS studies on primary and secondary metabolites have been employed to explore the metabolites - gene linkages properties in *Arabidopsis thaliana* and *O. sativa* that were used to validate through identification of novel molecular cross-talks between metabolites and genes^17,22,24,25^. In addition, mGWAS coupled with high-throughput computational (or cmGWAS) approaches were also used in studying the molecular interaction of metabolite synthesising genes and their relevant metabolism in AbS dynamics^26–28^. Though enough reports are available on several aspects for this well characterized model plant, to the best of our knowledge, no cmGWAS analysis has been carried out so far, despite the availability of high quality of reference genome and self-fertilization process^17,24^.

Taking this into consideration, we identified the Thr metabolite producing genes (ThrMPG) involved in Thr biosynthesis. Undefined potential of 16 ThrMPG have been predicted so far and these genes remain expressed in AbS. In addition, comparative mapping led to the identification of ThrMPG and their synteny in C4 grass genomes. These genes are to be studied further to characterize and delineate their molecular interventions in AbS signaling and metabolic engineering for the production of C4 rice plants with an improved nutrient traits and AbS tolerance.

## Results

### *In silico* identification of ThrMPG

**Figure 1:**
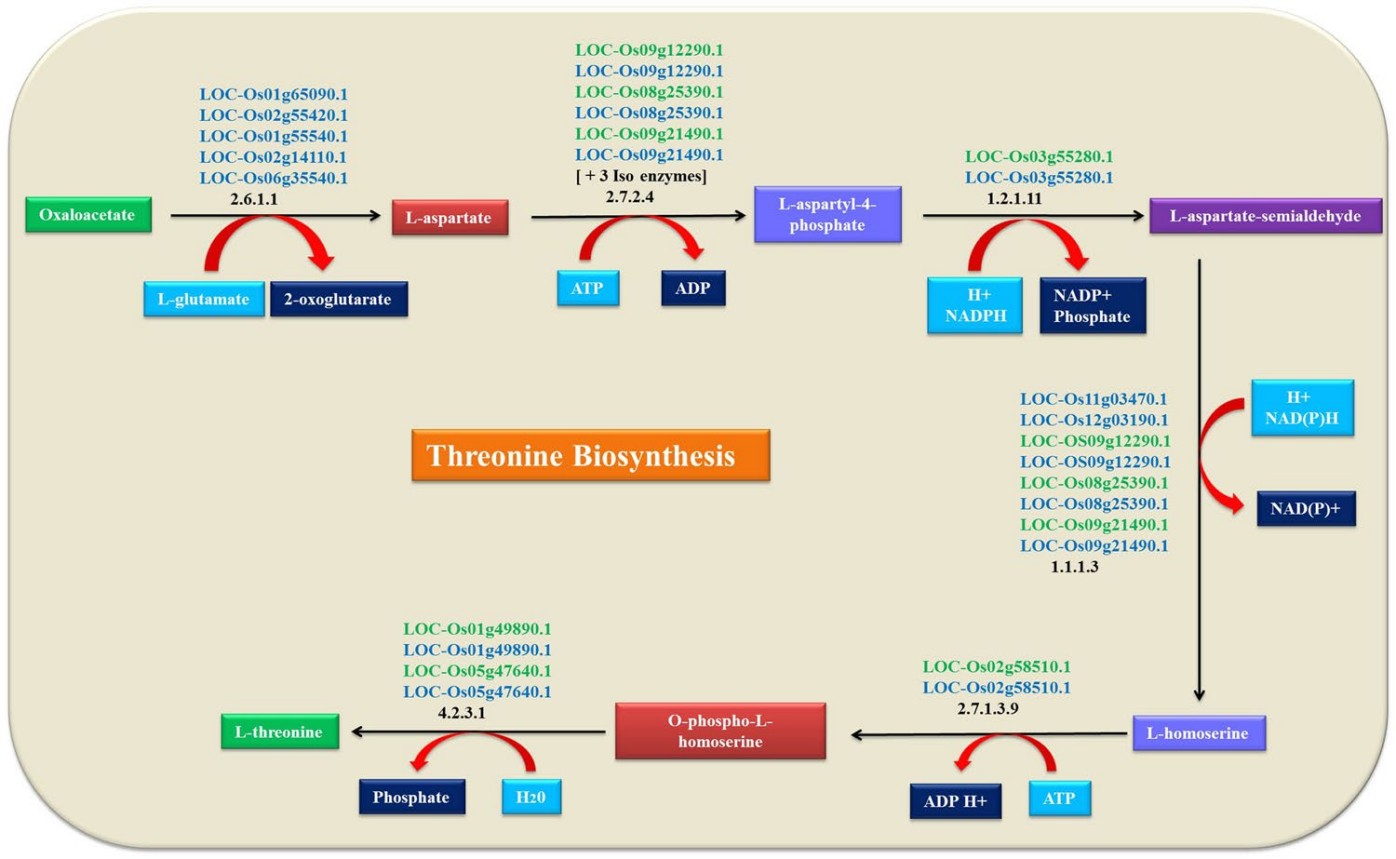
Threonine biosynthesis pathway.

Threonine (Thr) metabolite was used as a query in plant metabolic network database to retrieve the metabolite producing genes (MPG) and their biosynthesis pathway (Fig. 1). Sixteen abiotic stress responsible (AbSR) - ThrMPG genes that are involved in abiotic stress dynamics were identified (Table 1). Further, genomic, CDS, and protein sequences were retrieved via MSU - RGAP database.

**Table 1:** Threonine metabolite encoding genes.

S. No	Enzyme No	Enzyme name	Gene name	Locus name	Probe set ID
1	2.6.1.1	Aminotransferase,	Os01g0871300	LOC_Os01g65090.1	Os.7646.1.S1_a_at
2	2.6.1.1	Aminotransferase	Os02g0797500	LOC_Os02g55420.1	Os.18547.1.S1_at
3	2.6.1.1	Aminotransferase	Os01g0760600	LOC_Os01g55540.1	Os.9.1.S1_s_at
4	2.6.1.1	Aminotransferase	Os02g0236000	LOC_Os02g14110.1	Os.4167.1.S1_at
5	2.6.1.1	Aminotransferase	Os06g0548000	LOC_Os06g35540.1	Os.4772.1.S1_at
6	2.7.2.4	Bifunctional aspartokinase/homoserine dehydrogenase, chloroplast precursor	Os09g0294000	LOC_Os09g12290.1	Os.4417.1.S1_at
7	2.7.2.4	Bifunctional aspartokinase/homoserine dehydrogenase, chloroplast precursor	Os08g0342400	LOC_Os08g25390.1	Os.18966.2.S1_at
8	2.7.2.4	Bifunctional aspartokinase/homoserine dehydrogenase, chloroplast precursor	—	LOC_Os09g21490.1	OsAffx.29962.1.S1_at
9	1.2.1.11	Semialdehyde dehydrogenase	Os03g0760700	LOC_Os03g55280.1	Os.10389.1.S1_at
10	1.1.1.3	Bifunctional aspartokinase/homoserine dehydrogenase	Os11g0128800	LOC_Os11g03470.1	—
11	1.1.1.3	Bifunctional aspartokinase/homoserine dehydrogenase	Os12g0125400	LOC_Os12g03190.1	—
12	1.1.1.3	Bifunctional aspartokinase/homoserine dehydrogenase	Os09g0294000	LOC_Os09g12290.1	Os.4417.1.S1_at
13	1.1.1.3	Bifunctional aspartokinase/homoserine dehydrogenase	Os08g0342400	LOC_Os08g25390.1	Os.18966.2.S1_at
14	2.7.1.39	GHMP kinases	Os02g0831800	LOC_Os02g58510.1	Os.27193.1.S1_at
15	4.2.3.1	Threonine synthase	Os01g0693800	LOC_Os01g49890.1	Os.27749.1.S1_at
16	4.2.3.1	Threonine synthase	Os05g0549700	LOC_Os05g47640.1	Os.9175.1.S1_at

### AbSR - ThrMPG and their properties

The chromosome number, coding sequence, nucleotide and amino acid length, molecular weight, isoelectric point, subcellular localization and UniProt accession ID of the AbSR - ThrMPG were analyzed and are given in Table 2.

**Table 2:** Gene attributes of threonine.

S. No	Locus name	Chr. No	CDS	Nt L	aa L	M. Wt	pI	SL	UniProt ID
1	LOC_Os01g65090.1	1	37779509–37782884	1377	459	48908.2	7.9117	Ct	Q5N9Z8
2	LOC_Os02g55420.1	2	33942024–33946395	1377	459	50350.6	8.2736	Ct	Q6KAJ2
3	LOC_Os01g55540.1	1	31998651–32003690	1383	461	49934	8.7017	Cyto	P37833
4	LOC_Os02g14110.1	2	7706619–7711598	1299	433	48107.9	8.0795	Mt	Q6EUS6
5	LOC_Os06g35540.1	6	20727092–20731771	1392	464	51320.9	8.3875	Mt	Q0DBN4
6	LOC_Os09g12290.1	9	6995049–7009516	2748	916	99664	7.3922	Ct	Q69LG7
7	LOC_Os08g25390.1	8	15438856–15450607	2766	922	99973.2	6.8229	Ct	Q0J6A9
8	LOC_Os09g21490.1	9	12986634–12988418	645	215	24184.8	11.3632	N	—
9	LOC_Os03g55280.1	3	31460384–31463608	1128	376	40177.9	7.2219	Ct	Q93Y73
10	LOC_Os11g03470.1	11	1329410–1333415	1188	396	41901.2	6.3617	Ct	Q2RB23
11	LOC_Os12g03190.1	12	1216707–1220583	1155	385	40844.9	6.3623	Mt	Q2QYB7
12	LOC_Os09g12290.1	9	6995049–7009516	2748	916	99664	7.3922	Ct	Q69LG7
13	LOC_Os08g25390.1	8	15438856–15450607	2766	922	99973.2	6.8229	Ct	Q0J6A9
14	LOC_Os02g58510.1	2	35772233–35773369	1137	379	37812.4	7.2858	Ct	Q6K969
15	LOC_Os01g49890.1	1	28653963–28655948	1578	526	57702.6	6.6961	Ct	Q0JK67
16	LOC_Os05g47640.1	5	27290558–27292585	1566	522	57216.2	7.207	Ct	Q6L4H5

Chr, Chromosome; CDS, Coding sequence; Nt L, Nucleotide length; aa L, amino acid length; M.Wt, Molecular weight; pI, Isoelectric point; SL, subcellular localization; Cyto, Cytosol; N, Nucleus; Mt, Mitochondria; Ct, Chloroplast.

### Phylogenetic classification of ThrMPG

**Figure 2:**
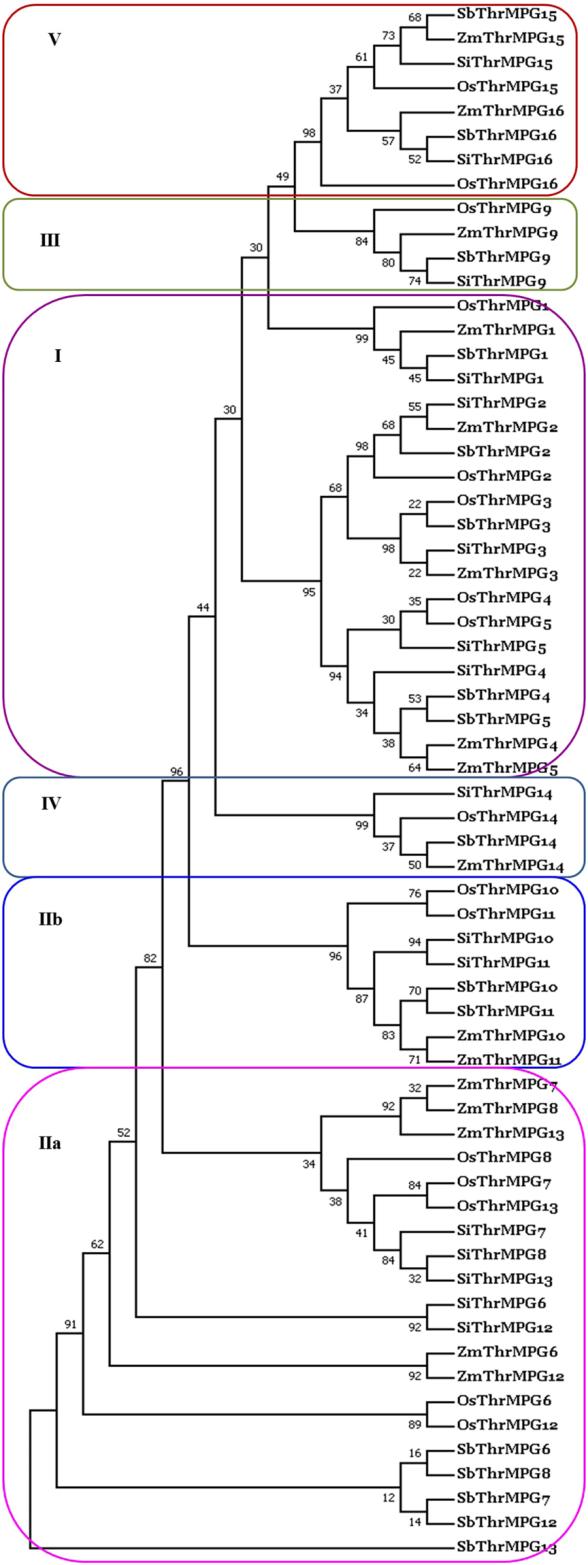
Phylogenetic tree relationship among AbSR –ThrMPG encoding proteins of rice, foxtail millet, sorghum and maize. Full length amino acid sequences of *Oryza sativa* (OsThrMPG), *Setaria italica* (SiThrMPG), *Sorghum bicolor* (SbThrMPG), and *Zea mays* (ZmThrMPG) were aligned by Clustal W and an unrooted maximum likelihood tree constructed with 1000 bootstrap iterations. Number at nodes represents the bootstrap values of the consensus tree obtained. The phylogenetic tree classified the ThrMPG proteins into (I–V) groups/classes.

The imputed protein sequences were used to generate the unrooted phylogenetic tree to study the phylogenetic organization of ThrMPG (Fig. 2). The unrooted tree divided the AbSR –ThrMPG into 5 major groups (I–V) based on the conserved Aminotran_1_2 (Aminotransferase class I and II), Homoserine _dh (Homoserine dehydrogenase), semialdehyde_dhC (Semialdehyde dehydrogenase C terminal domain), GHMP kinases_N (GHMP kinases N terminal domain) and PALP (Pyridoxal-phosphate dependent enzyme) domain and homology of ThrMPG encoding protein sequences (Supplementary Table 1). Group – I to V proteins have Aminotran_1_2, homoserine_dh, semialdehyde_dhC, GHMP_Kinases_N, PALP domains respectively. Group – II proteins are further classified into two subgroups (IIa and IIb) based on the homology and conservation of amino acid. Among the 64 annotated ThrMPG encoding proteins, 20 proteins belong to group – I, 28 to group – II, 4 to group – III, 4 to group – IV and 8 to group – V (Fig. 2). Phylogenetic analysis of group I–V proteins of OsThrMPG, SiThrMPG, SbThrMPG, and ZmThrMPG confirmed the group wise classification and also enabled the sub-classification of group – II proteins (Fig. 2). Among the 28 group – II ThrMPG proteins, 20 belong to IIa and 8 to IIb. Hence, based on our analysis, we infer that this phenomenon could be attributed to a special gene expansion event (obtained or lost) that could have occurred during the evolutionary process.

### Developmental tissues specific expression profiling of AbSR - ThrMPG

Of the 16 AbSR – ThrMPG, 14 genes exhibited tissue and stress specific expression profiling on diverse (41) rice plant developmental tissues. Of which, LOC_Os02g55420.1, LOC_Os01g55540.1, LOC_Os02g14110.1, LOC_Os08g25390.1, LOC_Os03g55280.1 and LOC_Os05g47640.1 showed a higher expression in various developmental tissues such as endosperm, embryo, young leaf, mature leaf, seed (S1 to S5), seedling (1–2 week old seedlings), shoot, flower and root as imputed by Rice DB which is dependent on the available AbSR – ThrMPG transcriptomic data (Supplementary Table 2). However, AbSR –ThrMPG revealed a negligible expression profiling in young inflorescence (P1–P6), ovary, stigma, coleoptile and root as imputed by Rice DB which is based on the available AbSR – ThrMPG transcriptomic data (Supplementary Table 2). Among 16 AbSR –ThrMPG, one gene (LOC_Os09g21490) had no expression in all 41 developmental tissues (Supplementary Table 2). AbSR – ThrMPG with functional annotation, experimentally shown motif, model sequences are given in Supplementary Table 3. Sixteen AbSR – ThrMPG and their co –expressed gene list are given in Supplementary Table 4.

### Phytohormonal expression profiling of AbSR-ThrMPG

**Figure 3:**
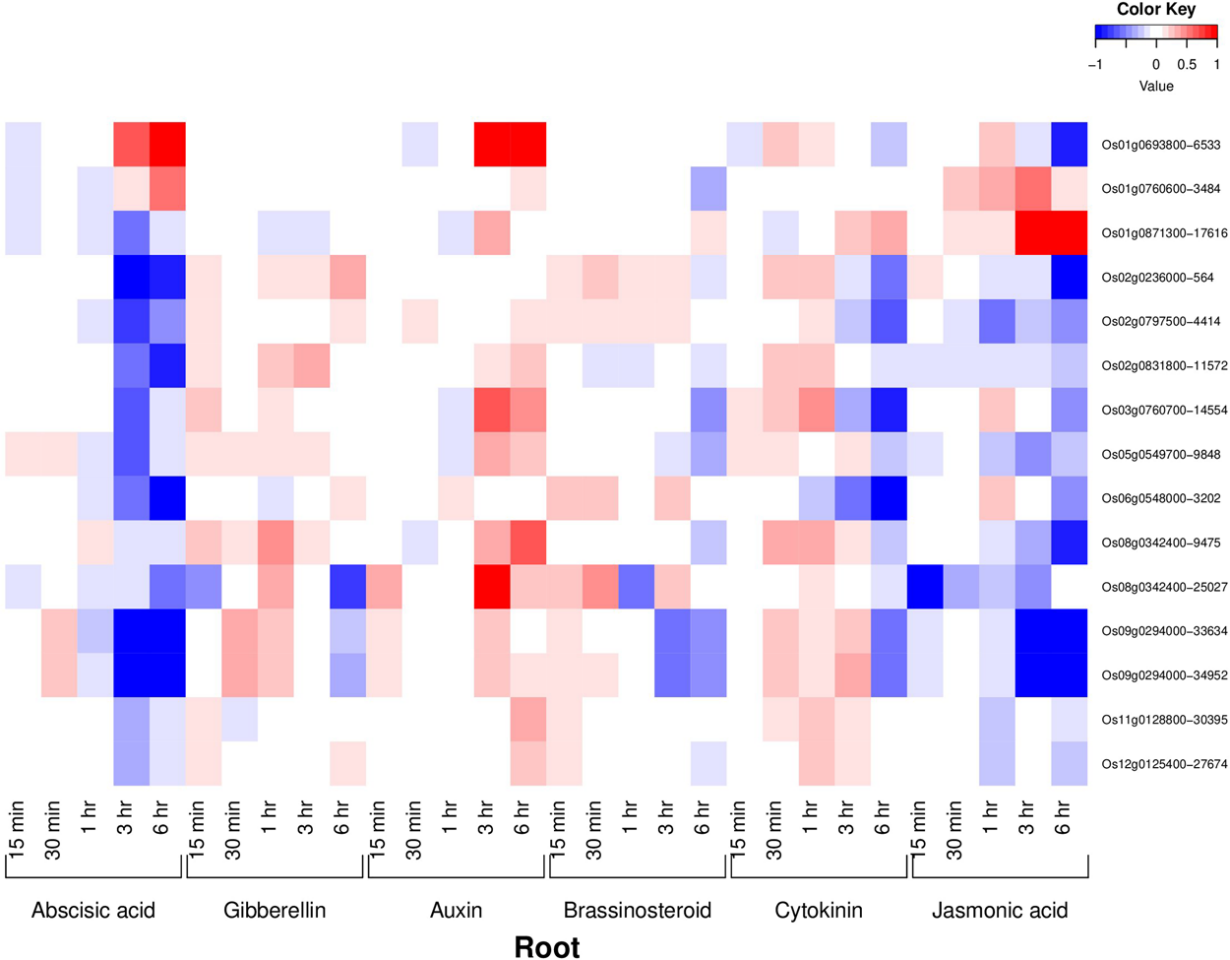
Heatmap representing the AbSR – ThrMPG and their phytohormonal expression profiling in root which are differentially expressed across the entire growth in the field conditions. Red color - up regulation; Blue color - down regulation; White color - unchanged. The colored scale bar at right side top denotes the relative expression value, where -1 and 1 represent down and up regulation of AbSR – ThrMPG.

**Figure 4:**
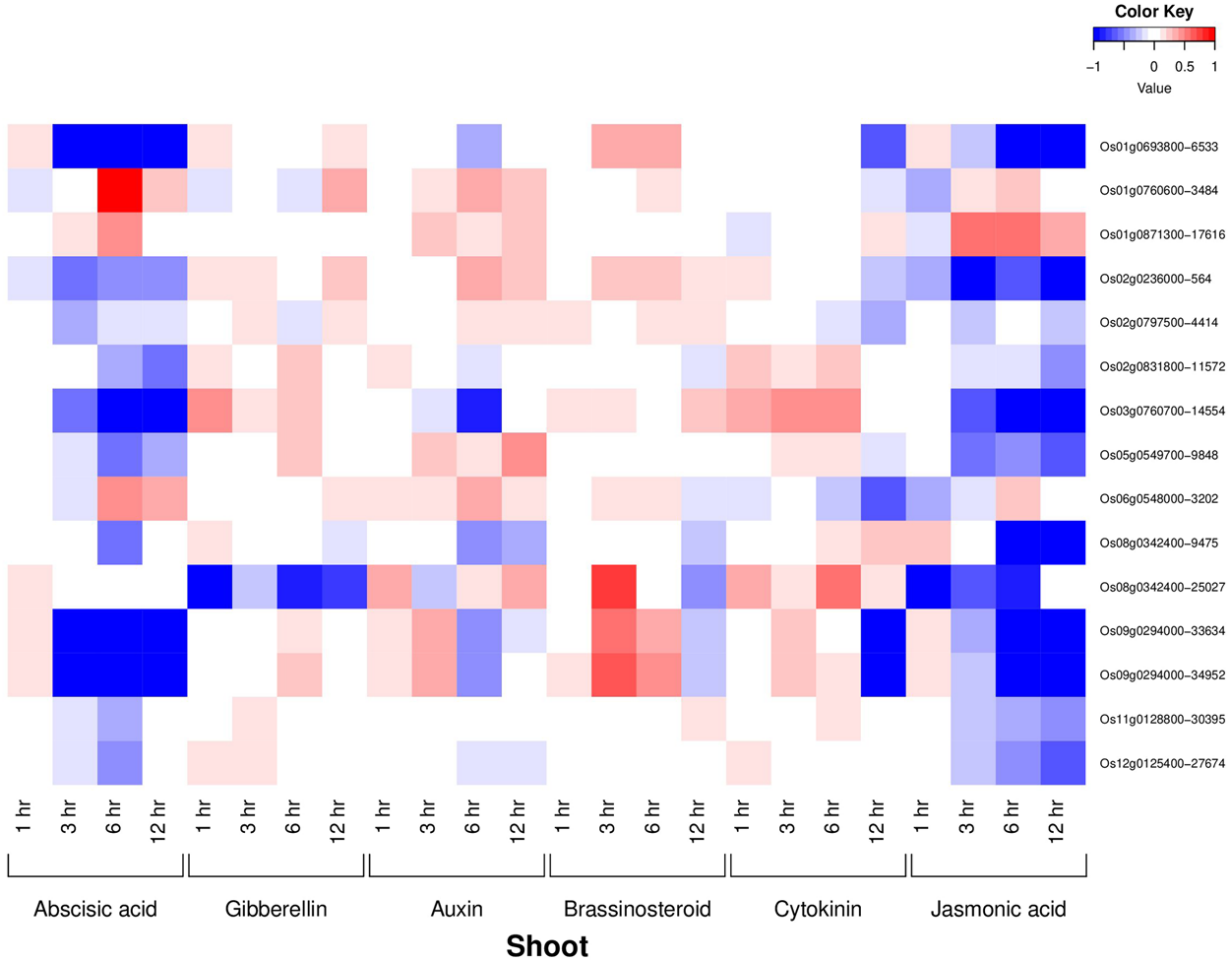
Heatmap representing the AbSR – ThrMPG and their phytohormonal expression profiling in shoot which are differentially expressed across the entire growth in the field conditions. Red color - up regulation; Blue color - down regulation; White color - unchanged. The colored scale bar at right side top represents the relative expression value, where -1 and 1 represent down and up regulation of AbSR – ThrMPG.

Among 16 AbSR-ThrMPG, 15 genes showed phytohormonal expression profiling in various time points such as 1 hr, 3 hr, 6 hr, 12 hr and 15 min, 30 min, 1 hr, 3 hr, 6 hr on shoot and root, respectively (Figs 3 and 4). In root, all the 15 AbSR-ThrMPG showed increased auxin and decreased abscisic acid, gibberellins, brassinosteroid, cytokinin, jasmonic acid hormone expression levels across all the time points (Fig. 3). In shoot, low level expression of abscisic acid and jasmonic acid and negligible level expression of gibberellins, auxin, brassinosteroid and cytokinin were noticed for the 15 AbSR-ThrMPG in all the time points (Fig. 4).

### Spatio – temporal gene expression analysis of AbSR – ThrMPG

**Figure 5:**
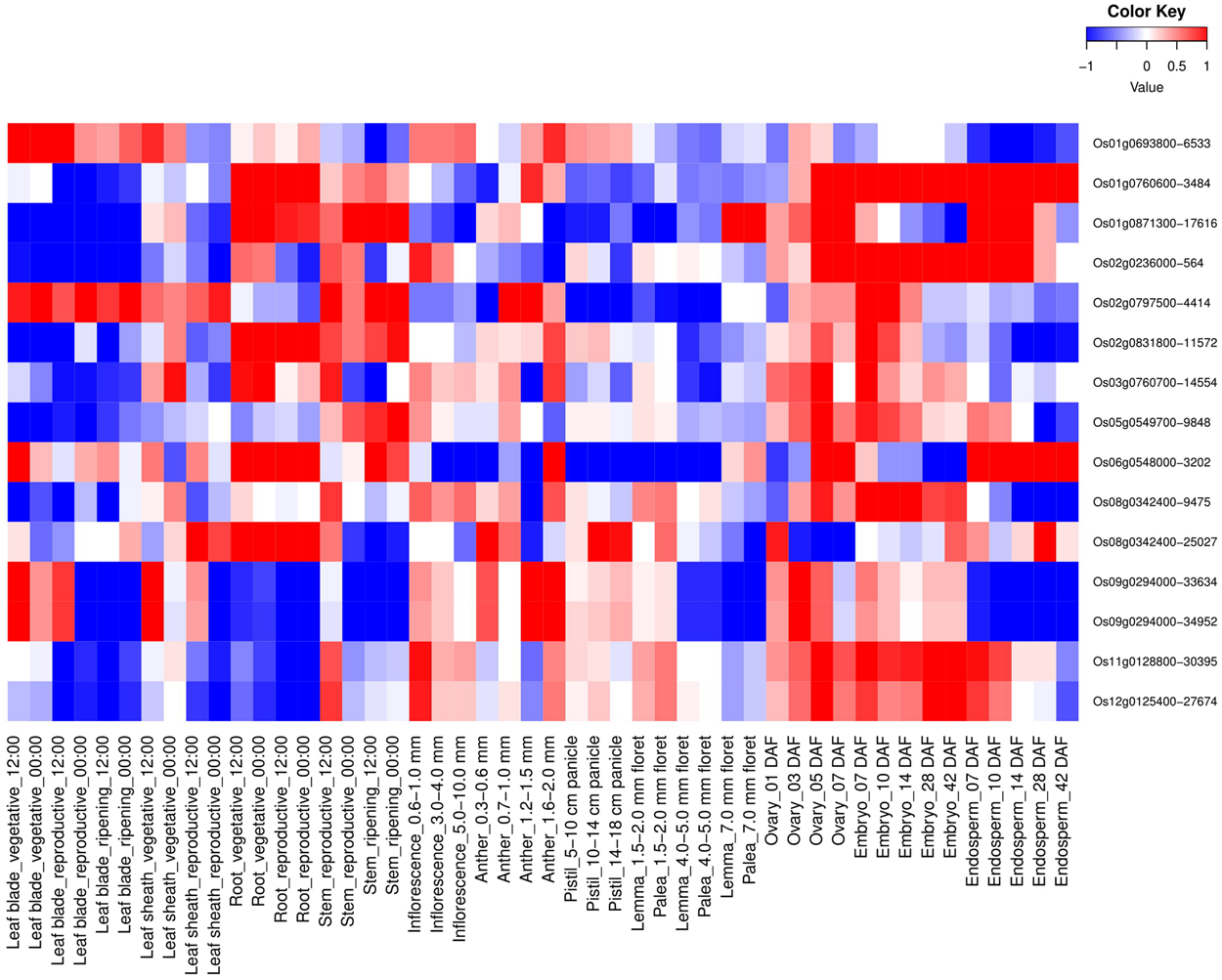
Heatmap representing the spatio – temporal expression profiling of tissues and organs at diverse developmental stages of AbSR –ThrMPG which are differentially expressed under natural field conditions. Red color - up regulation; Blue color - down regulation; White color - unchanged. The colored scale bar at right side top denotes relative expression value, where -1 and 1 represent down and up regulation of AbSR – ThrMPG.

Of the 16 AbSR – ThrMPG, spatio – temporal expression profiling was noticed for 15 genes in 48 different tissues/organs at diverse developmental stages. Few predominant genes such as Os01g076600, Os01g0871300, Os02g023600, Os02g0797500, Os03g0760700, Os06g0548000, Os11g0128800 and Os12g0125400 showed higher expression in diverse tissues and organs such as leaf blade (vegetative, reproductive, ripening), root (vegetative, reproductive), stem (ripening), ovary (05, 07 DAF), embryo (07, 10, 14, 28 and 42 DAF), endosperms (07, 10, 14, 28 and 42 DAF) (Fig. 5). AbSR – ThrMPG exhibited negligible expression in leaf sheath (vegetative, reproductive), stem (reproductive), inflorescence (0.6–1.0, 3.0–4.0, 5.0–10.0 mm), anther (0.3–0.6, 0.7–1.0, 1.2–1.5, 1.6–2. 0 mm), pistil (5–10, 10–14, 14–18 cm panicle), lemma (1.5–2.0, 4.0–5.0, 7.0 mm floret), palea (1.5–2.0, 4.0–5.0, 7.0 mm floret) and ovary (01, 03 DAF) (Fig. 5) as imputed by RiceXPro which is dependent on the available AbSR – ThrMPG field transcriptomic data (Supplementary Table 5).

### Conserved motif and SNP of AbSR - ThrMPG

The identified 16 AbSR – ThrMPG and their SNPs were searched based on the SNP by region and SNP within genes of *O. sativa* ssp*. Japonica*. A total of 1373 and 1028 SNPs were found in region (Supplementary Table 6) and within gene (Supplementary Table 7) specific SNPs respectively. Experimentally shown motif analysis of these AbSR – ThrMPG genes exhibited the presence of 481 motifs, of which few elements were found in all the 16 AbSR – ThrMPG, while some were unique to one or two genes of ThrMPG (Supplementary Table 8). CACGTG (ABRE binding site motif), ACCACG (NAC), CCACGT (CBF2 binding site motif), AAGTGA (SORLIP3), AAGAGC (ERE promoter motif), ACAACA (RAV1-A binding site motif), CTTGAC (W-box promoter motif), AAGCTT (HSEs binding site motif), CACGTA (ACE promoter motif), AGATAG (GATA promoter motif), GGCCGA (CBF1 BS in cor15a) were found in upstream region of 16 AbSR – ThrMPG. In contrast, few motifs were found in only one AbSR – ThrMPG (Supplementary Table 8). This include, GATAAG (Ibox promoter motif; in LOC_Os06g35540.1), ACGTGT (Z-box promoter motif; in LOC_Os05g47640.1), CAATTATTA (ATHB6 binding site motif; in LOC_Os02g55420.1; Supplementary Table 8).

### AbSR - ThrMPG gene interaction network

**Figure 6:**
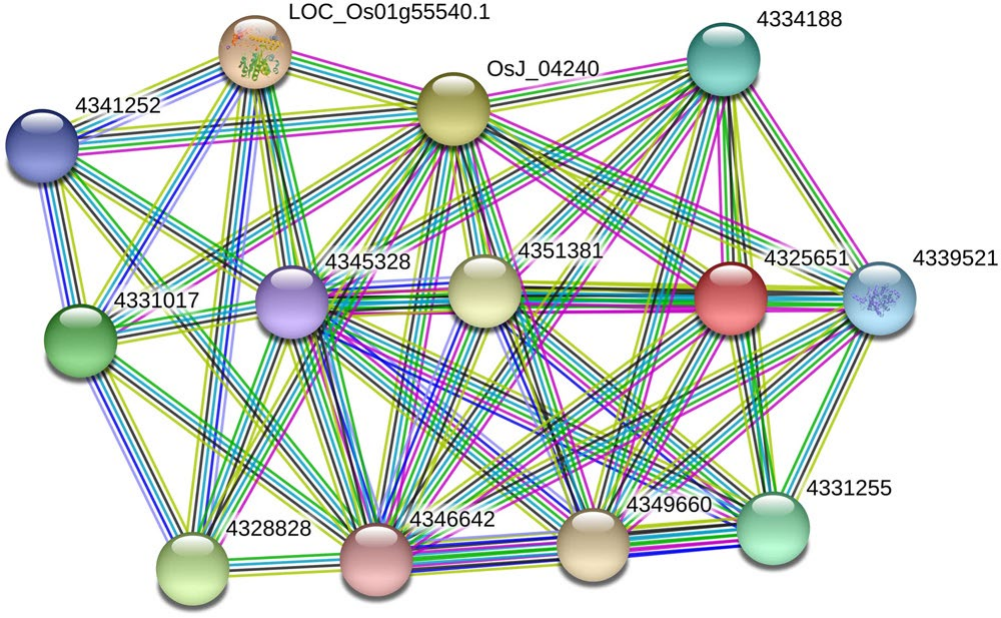
AbSR – ThrMPG molecular interaction network. *Japonica* rice – AbSR – ThrMPG interaction showing strongly connected functional modules. Colored lines between the proteins indicate various types of interaction evidence. Green color, gene neighborhood; red color, gene fusions; blue color, gene co-occurrence; black color, co-expression; sand green color, text-mining; pink color, experimentally determined; violet color, protein homology. Filled protein nodes that represent the availability of protein 3D structural information is known or predicted.

Sixteen threonine encoding seed proteins were derived from *O. sativa* ssp. *japonica* AbSR - ThrMPG gene interaction network analysis. The interaction network had 14 nodes and 51 edges (Fig. 6). The proteins of the AbSR – ThrMPG interaction had an average of 7.29 nodal degrees within the neighbour proteins. Protein – protein interaction (PPI) of AbSR - ThrMPG enrichment *P* – value was <0.01. This molecular network significantly interacted with more than expected based among the random set of proteins of similar size that existed in the genome. Such enrichment indicates biological connectivity among the interacting groups. In addition, the network exhibited the complexity of AbSR –ThrMPG, their interlinked relationships proving its multigenic nature.

### Structure of AbSR - ThrMPG

**Figure 7:**
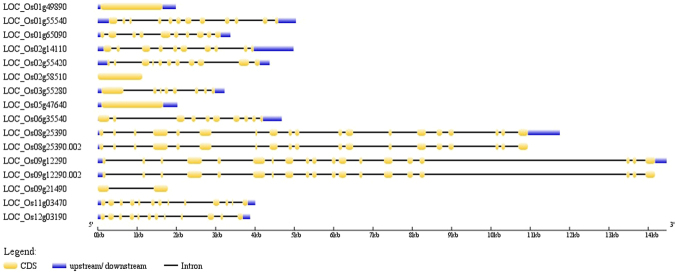
Gene organization of threonine encoding genes. The blue lines indicate the UTR regions, orange boxes indicate the exons and black lines show the introns.

Positions of exons and introns within the 16 AbSR - ThrMPG genes were imputed. Gene structure determination showed the numbers and arrangements of exons and introns (Fig. 7).

### GO annotation

**Figure 8:**
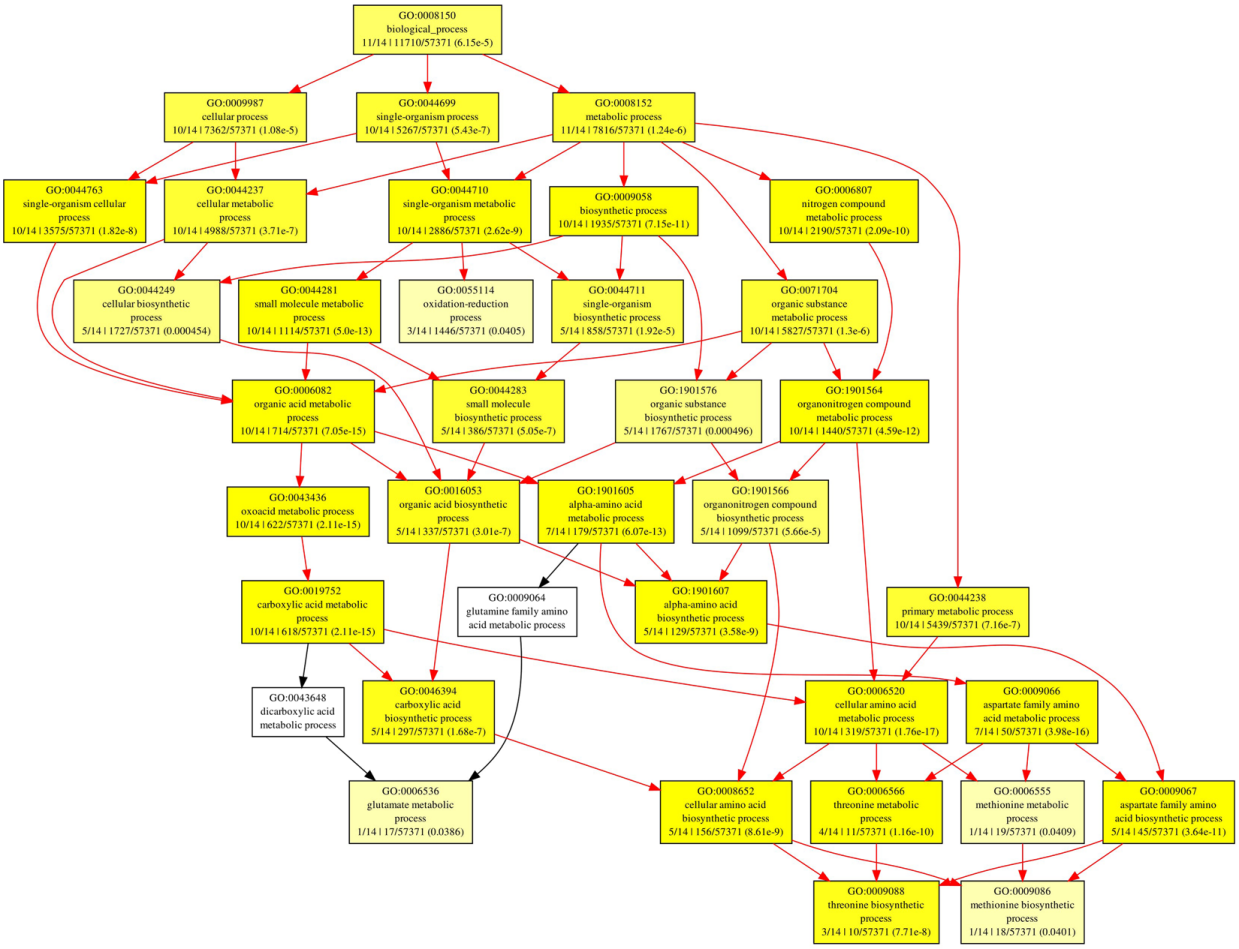
Classification of AbSR -ThrMPG based on their biological process. Boxes represent GO terms, term definition, labelled by its GO ID, and detailed information, organized as ‘q/m|t/k (*P*-value)’ (‘k’- genes from microarray; ‘t’- microarray with total of genes; ‘q’- genes within ‘k’; ‘m’– genes within ‘t’ associated with it. The GO enrichment analysis formula adopted from Zheng and Wang, 2008). Yellow color boxes indicate significantly enriched GO terms. The level of color saturation of each node is positively correlated to the significant level of the corresponding GO term. White color boxes represent non – significant GO terms within the hierarchical tree. Arrows signify the connections between different GO terms. Red color arrows indicates relationships between two enriched GO terms, black color arrows represent relationships between enriched and un-enriched GO terms.

**Figure 9:**
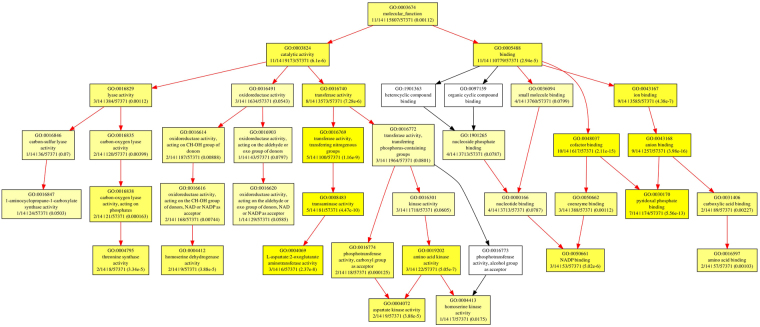
Classification of AbSR -ThrMPG based on their molecular function. Boxes represent GO terms, term definition, labelled by its GO ID, and detailed information, organized as ‘q/m|t/k (*P*-value)’ (‘q/m|t/k’- GO term enrichment analysis values calculated by Fisher’s exact test. The GO enrichment analysis formula adopted from Zheng and Wang, 2008). Yellow color boxes indicate significantly enriched GO terms. The level of color saturation of each node is positively correlated to the significant level of the corresponding GO term. White color boxes represent non – significant GO terms within the hierarchical tree. Arrows signify the connections between different GO terms. Red color arrows indicates relationships between two enriched GO terms, black color arrows represent relationships between enriched and un-enriched GO terms.

Gene characteristic features of AbSR - ThrMPG were analysed by probeset IDs using GOEAST and showed significant involvement of these proteins in different molecular functions and biological processes. The AbSR- ThrMPG encoding proteins were predicted to be involved in diverse cellular, metabolic and biosynthetic processes. The results are presented in Fig. 8. The inherent molecular functions of these proteins corresponded to different types of catalytic activity, cofactor and ion binding activity (Fig. 9**)**.

### Comparative mapping in C4 grass species

**Figure 10:**
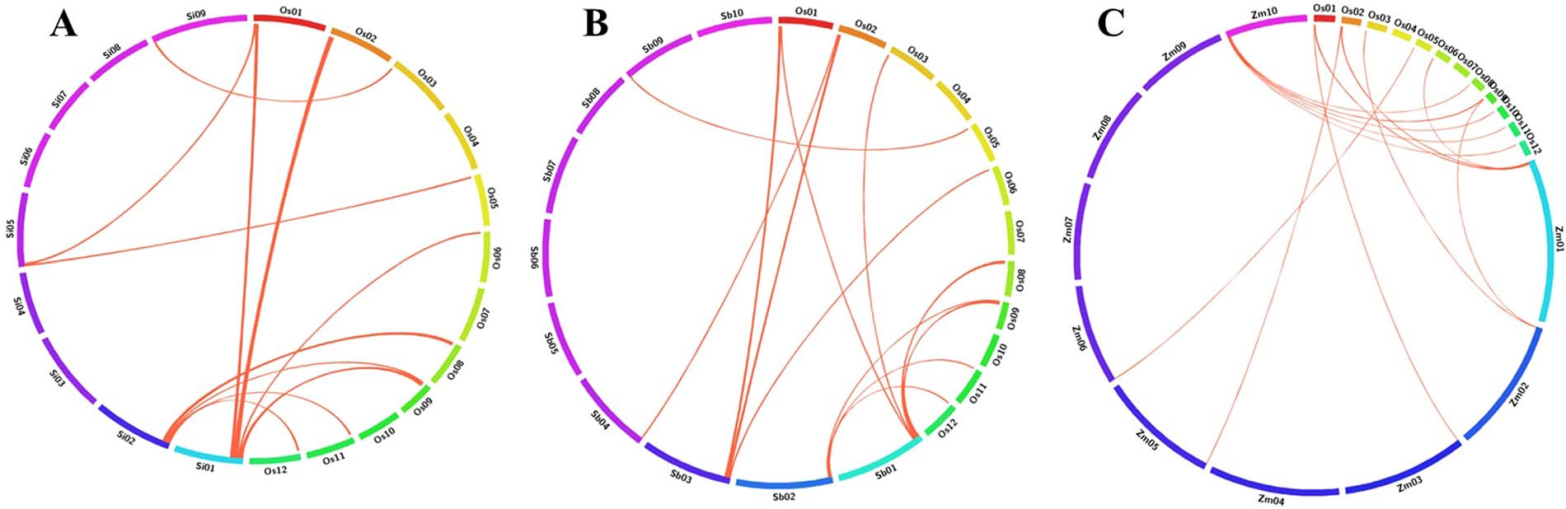
Orthologous relationships between threonine-encoding genes of (**A**) rice and foxtail millet, and (**B**) rice and sorghum, and (**C**) rice and maize. Each segment indicates chromosome and the orthologous genomic regions are marked with red.

BLAST search analyses assumed the chromosomal colinearity among the 16 AbSR - ThrMPG of rice (*japonica*) in comparison with its related (C4) grass genomes of *S. bicolor*, *Z. mays*, and *S. italica* by chromosomal localization and were compared with their respective chromosomes. The comparative mapping depicted maximum synteny between *O. sativa* and *S. bicolor*, *O. sativa* and *Z. mays*, followed by *O. sativa* and *S. italica* [16 AbSR - ThrMPG (~100%)] (Fig. 10A–C; Supplementary Tables 9–11).

## Discussion

Metabolites especially amino acids have diverse pivotal functions in plants. They are mainly involved in signaling processes, protein biosynthesis and stress responses in plants. Generally, the 20 standard amino acids strongly vary, substantially and dynamically change based on the environmental factors, physiological and developmental stage of the plant cell^29^. Though biosynthesis of amino acids has been examined in detail, but only little attention has been paid so far to regulate their pool size, catabolism of amino acids, and identification of metabolites producing genes. We focus elaborately on the identification of threonine metabolite producing genes (ThrMPG) and their biosynthesis pathway. Threonine plays an essential role in accepting the signals from receptors that sense the phytohormones, adverse environmental conditions, and other physicochemical factors, and translating it into specific functional outputs such as plant growth, development, and changes in metabolism, gene expression, seed development, storage protein gene expression, abiotic stress tolerance, cell growth and division^13–16,30^. In addition, two major enzymes are essentially involved in threonine biosynthesis namely aspartate aminotransferase (AAT) and bifunctional aspartokinase/ homoserine dehydrogenase (AK-HSD). AAT (EC 2.6.1.1) is an enzyme significantly involved in various plant physiological processes, namely - the amino acid biosynthesis, regulating carbon and nitrogen metabolism^31^, recycling the carbon skeleton during ammonia absorption in roots^32^, hiring asparagine nitrogen during grain filling^33^ intracellular carbon shuttles in C4 plants^34^. AK-HSD (EC 1.1.1.3 and 2.7.2.4) is an essential enzyme that participates in the improvement of nutritional value in food crops especially in rice^35–38^. Besides, threonine is an essential (amino acid) metabolite easily interconnected with methionine, isoleucine and interconverted with glycine, serine^29,30,39,40^. There is still lack of information about the importance of threonine metabolite mechanisms, its alternative metabolic pathway and its accumulation under AbS. Hence, we focused on threonine metabolite for understanding the molecular cross talks of ThrMPG, hormonal dynamism, physiological and physicochemical properties.

Our study was the first to report cmGWAS of ThrMPG analysis in rice which is an ehrhartoideae C3 model crop and also panicoid C4 grass species. In view of the significance of abiotic stress biology of rice, the present study was undertaken to identify and characterize ThrMPG using *in silico* approaches and examine their tissues specific expression patterns, plant hormonal dynamism, transcriptional regulation, physiological and physicochemical properties in response to multiple abiotic stress (AbS).

Our research highlights the importance of cmGWAS of ThrMPG in rice, wherein 16 ThrMPG were identified from plant metabolic network analyser. The ThrMPG were retrieved and subjected to MSU- RGAP and RiceNetDB to study the metabolic biosynthetic pathway. These 16 OsThrMPG remain exposed into Rice DB to have better insights on developmental tissues specific expression of genes. Further, based on our analysis we infer that these 16 OsThrMPG are likely to be involved in different abiotic stress (AbS). In addition, these abiotic stress responsible (AbSR) - ThrMPG were chosen for expression profiling under various individual AbS. Available public microarray hybridization DB of rice and their respective expression values confirmed the developmental tissue specific expression profiling of 16 AbSR – ThrMPG. The plants are simultaneously affected by stress in diverse developmental tissues^41–43^. Therefore, these 16 AbSR-ThrMPG reveal higher levels of AbSR - ThrMPG expression and thus a similar expression pattern in 41 different developmental tissues at different individual AbS. Hence, our analysis suggests their diverse role in different molecular and physiological activities. For delineating their functional roles, this information may be used to choose potential candidate genes based on their improved expression patterns^43^. This data could be further used to enhance our understanding on rice metabolism and to improve the quality and quantity of rice grain by developing AbS tolerance plant through gene manipulation and genetic engineering.

All the genes related to the signal transduction and plant hormone metabolisms control agriculturally important traits determining the growth, development and crop yield^44^. In this pilot study, global 16 AbSR - ThrMPG were chosen for phytohormonal gene expression profiling in natural field conditions. Public repository of expression DB of rice and their corresponding expression dataset confirmed the plant hormonal expression of 15 out of 16 AbSR – ThrMPG based on the available transcriptomic datasets. Data analysis revealed the low/negligible level of expression of these 15 AbSR – ThrMPG for phytohormones such as abscisic acid, gibberellins, auxin, cytokinin, jasmonic acid and brassinosteroid in shoot and root of the rice plant at various time points. The level of amino acids as metabolite is generally increased in several plant species, particularly in rice, under AbS condition and they act as osmolytes^29,30,40^. Our analysis revealed that in natural field condition, abscisic acid and jasmonic acid are low level. However, elevated level is noticed in stress conditions as these two plant hormones play a significant role in abiotic and biotic stress conditions^45^ which can be considered as a phytohormonal expression signature. Thus, our plant hormone expression profile provides the molecular insights in understanding the expression signature of the AbSR – ThrMPG in combination with pattern in the field – development era and also in AbS conditions.

Spatio – temporal expression analysis of 16 AbSR – ThrMPG showed the differential expression signature in the 48 different tissues/organs and developmental stages implying the higher expression level of AbSR –ThrMPG from diverse tissues and organs and their expression in throughout the entire growth in the field. Taken together, spatio- temporal expression profiling in tissues and organs (field/development category) reveal the baseline information of AbSR –ThrMPG and their biological processes in the natural field condition^44^. On the other hand, the candidate genes provide the strong base for conducting overexpression studies in diverse tissues/organs in order to enhance the AbSR protein and improvement of nutritional content in rice.

To analyse the evolutionary relationship of *Oryza sativa* ThrMPG (OsThrMPG), a fair phylogenetic tree comprising ThrMPG from *Setaria italica* (SiThrMPG), *Sorghum bicolor* (SbThrMPG), *Zea mays* (ZmThrMPG) was constructed. The location of ThrMPG domains in OsThrMPG, SiThrMPG, SbThrMPG and ZmThrMPG was investigated employing domain analysis tool, namely HMMSCAN. The analysis showed that the distribution of phylogenetic groups corresponds well with the domains and sequence conservation. Results revealed that the imputed protein sequences are closely related to C4 grass species and exist as a chromosomal colinearity in foxtail millet, sorghum, maize.

SNPs are majorly responsible for genomic variations in plants and have been used as important molecular markers in genetics studies. SNPs are highly associated with metabolites and it regulates the agronomical traits^46–48^. We evaluate the metabolite – SNP pairs in rice and determine the functional regulatory relationship by evaluating the similarities in the changes of enzymes in a particular pathway. In addition, metabolite – SNP pairs highly associated with significant agronomical traits, such as the plant architecture, yield component, disease resistance, flowering time, and abiotic stress tolerance^46^. Our new findings confirmed the similarities in the enzyme modulatory function of the ThrMPG pathway that may be useful for metabolite - SNPs association studies.

Comparative mapping of AbSR - ThrMPG and their responsive proteins on *O. sativa* (Os), *S. bicolor* (Sb), *S. italica* (Si), and *Z. mays* (Zm) databases were imputed to comprehend the orthologous relationships between the C4 grass species genomes. OsThrMPG showed maximum synteny with Sb, Si, and Zm genes (∼100%) due to their wide range gene level synteny. Our analysis clearly states that OsThrMPG has been found to be homologous to SbThrMPG, SiThrMPG, and ZmThrMPG respectively and its close evolutionary associations revealed the novel insights about C3 and C4 crop species. This comprehensive information on the comparative map could serve as better source for the evolutionary process of ThrMPG amid the Gramineae members.

In order to elucidate the regulatory functions of AbSR -ThrMPG in various stress responses by searching the *cis* – elements against (experimentally shown motif) the Rice DB, we identified putative AbSR *cis*-elements in the 1-kb promoter regions upstream of the transcription start codon (ATG) of AbSR –ThrMPG. Various types of *cis*-elements, which are directly related to the dehydration-responsive element (DRE)^49^, or ABA-responsive element (ABRE)^49,50^, WRKY^51^, NAC^52^, heat shock elements (HSEs)^53^, and C-repeat (CRT)/DRE motif-binding transcription factors (CBFs)^54,55^.

AbSR - ThrMPG, their own protein interactions, and functional relationships exploited the complexity of AbS upon neighbourhood protein modules and the conformation of its nodes, genes and their connecting edges were expressed in AbS studies^43^. These AbSR-ThrMPG corresponding proteins have maximum variation in length of amino acids, molecular weight, isoelectric point values of these proteins. Additionally, subcellular localization of these proteins at independent organelles may be attributed to the presence of putative novel variants, which are prerequisite for further validation.

AbSR - ThrMPG encoding proteins seems to have a pivotal role in diverse developmental processes and AbS response in plants, especially in rice. To date, the function and related regulatory mechanism of these (ThrMPG encoding) proteins in rice and other grass genomes remain poorly understood. Therefore, the computational analysis of the ThrMPG provides an important reference for future studies on the biological functions of rice ThrMPG encoding proteins. In addition, this study provides not only an annotation of the ThrMPG in rice, but also identified the novel functions of ThrMPG with respect to cold, salinity, drought and heat stresses. Importantly, our findings provide an invaluable and essential functional reference for rice ThrMPG and to study the regulation of unique and combined AbS response.

## Conclusion

The use of an ultra- high throughput computational omics approaches have led to the development and application of rice cmGWAS. This novel analysis method provides an understanding of transcriptional regulation, metabolic response of rice to diverse physiochemical environmental conditions, and also in pivotal relation to AbS. Metabolite producing gene regulations have been well investigated in all major food crops and as well as tree species. To the best of our knowledge, no such study on AbSR – ThrMPG has been conducted in *O. sativa*, to explore C3 photosynthesis, AbS resistance mechanism and C4 rice plant production. Considering the significance of rice crop and AbSR – ThrMPG, the present study has used high throughput computational approaches to ascertain and annotate the AbSR – ThrMPG. The recognized genes were used for the gene ontology annotation, phylogenetic analysis, comparative mapping, and gene structures, and gene interaction, phytohormonal expression followed by motif and SNP imputations. Thus, the computational expression pattern of rice AbSR – ThrMPG was extensively analysed to understand the expression profiling of genes in multiple developmental tissues which helped in unravelling the functional regulation under AbS responses in rice.

The current study hypothesize that AbSR - ThrMPG encoding proteins may activate the many downstream genes which are involved in osmolytes regulation and these are yet to be characterized. Osmolytes play a pivotal role in AbS avoidance and tolerance mechanisms. Osmolytes such as glycine, lysine, proline, sucrose, betaine, fructose, mannitol and myo-inosital enhance the oxidative stress mechanism by scavenging the ROS molecules and simultaneously provide AbS endurance by changing the physiological, molecular and signal transduction processes in plants.

The bioinformatics expression analysis depends on the publicly available microarray datasets from experiments conducted with rice to decipher the transcriptional regulation of AbSR-ThrMPG in amino acid biosynthesis and AbS mechanism. These analyses were performed to understand the role of AbS in abscisic acid (ABA) dependent and independent manner and interfere the regulation of quenching ROS responsible genes. However, advanced studies are needed to understand the detailed mechanism of action of the AbSR-ThrMPG under several AbS. Furthermore, the emerging technologies such as NGS based RNA-seq needs whole genome sequenced models to make transcriptomic data in higher quality. Combinatorial omics (transcriptome, epigenome, proteome, hormanome and metabolome) approaches, systems biology, mathematical modelling will aid to unravel the metabolite and or amino acid biosynthesis, signaling and AbS regulatory mechanisms. It will pave the way for metabolic engineering and over-expression studies. Further, extension of the present study using food crops such as rice, foxtail millet, maize, sorghum and also in *Arabidopsis thaliana* is expected to throw more glows on the key candidate genes function and their metabolism in the enhancement of nutrition and development of AbS tolerance.

## Materials and Methods

### Identification of metabolite producing genes

Rice abiotic stress responsible (AbSR) metabolite like threonine (Thr)^13,14^ was subjected to OryzaCyc database available in plant metabolic network database (http://plantcyc.org/) and identified and retrieved the AbSR – Thr metabolite producing genes (AbSR-ThrMPG) as shown in Table 1.

### Mining of gene attributes

AbSR - ThrMPG were subjected to RiceNetDB (http://bis.zju.edu.cn/ricenetdb/) to retrieve the chromosome number, coding sequence, nucleotide and amino acid length, molecular weight, isoelectric point, subcellular localization and UniProt accession ID for further protein functional analysis as shown in Table 2.

### Database searches for ThrMPG and Phylogenetic analysis

*Oryza sativa* ThrMPG (OsThrMPG) protein sequences were retrieved in an exhaustive manner from the RGAP (http://rice.plantbiology.msu.edu/)^56^. OsThrMPG genes in three sequenced C4 grasses foxtail millet (*Setaria italica*), sorghum (*Sorghum bicolor*), maize (*Zea mays*) were also identified by BLASTP (https://blast.ncbi.nlm.nih.gov/Blast.cgi?PAGE=Proteins) analysis of the protein sequences, against these grass species. The accession numbers of newly identified ThrMPG from foxtail millet, sorghum and maize which were named as SiThrMPG, SbThrMPG and ZmThrMPG, respectively, along with the % of similarity are listed in Supplementary Table 12. The identified ThrMPG sequences were confirmed for the presence of PFAM domain using HMMSCAN (http://www.ebi.ac.uk/Tools/hmmer/search/hmmscan) and listed in Supplementary Table 1. The OsThrMPG sequences along with SiThrMPG, SbThrMPG, ZmThrMPG were imported to MEGA v7.0^57^ to construct a phylogenetic tree by maximum - likelihood method and the bootstrap test was performed with 1000 iterations.

### Analysis of developmental tissues specific expression

AbSR – ThrMPG and their corresponding locus IDs were exposed to Rice DB^42^. Developmental tissue specific expressions with their functional regulations were analysed based on the retrieved data. All RNA expression data was exposed to 75 percentile normalization and log2 transformation using in – house R program available in RiceXPro^44,58^ integrated with Rice DB. All the AbS –ThrMPG were subjected to PLANEX DB (http://planex.plantbioinformatics.org/co-expression) to retrieve the Pearson’s correlation coefficient (PCCs, r - value) with co-expressed genes along with their probeset IDs against *O. sativa*.

### Spatio – temporal and phytohormone expression analysis

AbSR - ThrMPG were exported to spatio-temporal (RXP_0001) dataset and plant hormone (RXP_1001~RXP_1012) datasets which are available in RiceXPro (http://ricexpro.dna.affrc.go.jp/)^44^ database for analyzing the spatio-temporal gene expression signatures of tissues/organs, and hormonal expression dynamism of shoot, root.

### Gene structure and SNP of AbSR -ThrMPG

The genomic sequences and coding sequences of 16 AbSR-ThrMPG were analysed by GSDS web server v2.0 (http://gsds.cbi.pku.edu.cn/)^59^ to identify the position of exons and introns. RAP–IDs or locus IDs of 16 ThrMPG were exposed to RiceVarMap v6.1 (http://ricevarmap.ncpgr.cn/)^47^ and selected the chromosome number; laid the range gene region to search for variations by region and variations by gene, respectively. Genomic region and within gene specific single nucleotide polymorphism (SNP) were identified and retrieved.

### Promoter analysis

AbSR – ThrMPG and their corresponding locus IDs were exposed to Rice DB^42^. The occurrence of all possible *cis*-acting elements and their expression regulation has been experimentally confirmed already and it matched in 1 kb upstream region of each ThrMPG in Rice DB integrated with AGRIS, matched in rice promoters. The *cis* elements, name, sequence and hyperlinked PubMed ID were identified and retrieved.

### Functional gene ontology (GO) analysis

Identified AbSR - ThrMPG (Table 1) and their corresponding probeset IDs of Affymetrix gene chips were subjected to GOEAST (http://omicslab.genetics.ac.cn/GOEAST/php/affymetrix.php)^60^ to obtain GO against rice array platform based on the Benjamini and Yekutieli false discovery correction value at 0.01 for the genes. GO enrichment was conducted using GOEAST and the input probeset IDs (gene) list was calculated by Fisher’s exact test^60^.

### Signaling network analysis

The protein – protein interaction (PPI) of these annotated 16 AbSR – ThrMPG was done using STRING v10.5 with a high confidence score of 0.7. PPI enrichment analysis was done using 0.01 level of significance. The interactome was used to understand the physical and functional role of the player’s involved^61^.

### Comparative mapping of rice AbSR – ThrMPG in C4 grass genome

The amino acid sequences of physically mapped AbSR - ThrMPG were BLASTP searched against the peptide sequences of *Sorghum bicolor, Zea mays*, and *Setaria italica* (http://gramene.org/) to impute the corresponding orthologs in these grass species. All hits with minimum 60% homology remained treated as significant. The chromosomal colinearity between *O. sativa* and these C4 grass species were then pictured by using Circos v0.55 (http://circos.ca/)^62^.

## Electronic supplementary material

Dataset 1

Dataset 2

Dataset 3

Dataset 4

Dataset 5

Dataset 6

Dataset 7

Dataset 8

Dataset 9

Dataset 10

Dataset 11

Dataset 12
